# Blocking of carnitine palmitoyl transferase 1 potently reduces stress-induced depression in rat highlighting a pivotal role of lipid metabolism

**DOI:** 10.1038/s41598-017-02343-6

**Published:** 2017-05-19

**Authors:** Anne Skøttrup Mørkholt, Ove Wiborg, Jette G. K. Nieland, Søren Nielsen, John Dirk Nieland

**Affiliations:** 1 Aalborg University, Department of Health Science and Technology, Aalborg, 9220 Denmark; 2 Aarhus University, Department of Clinical Medicine, Risskov, 8240 Denmark; 3 Meta-IQ, ApS, Aarhus, 8000 Denmark

## Abstract

Major depressive disorder is a complex and common mental disease, for which the pathology has not been elucidated. The purpose of this study is to provide knowledge about the importance of mitochondrial dysfunction, dysregulated lipid metabolism and inflammation. Mitochondrial carnitine palmitoyl transferase 1a (CPT1a) is a key molecule involved in lipid metabolism and mutations in CPT1a causing reduced function is hypothesized to have a protective role in the development of depression. Moreover, CPT1a is found to be upregulated in suicide patients with history of depression. Therefore, we hypothesized that inhibition of CPT1a activity can be developed as an innovative treatment strategy for depression. Stress exposure combined with different pharmacological treatment regimens; Etomoxir, CPT1 blocker, and Escitalopram, a favoured antidepressant drug, was applied in state-of-the-art chronic mild stress model. Etomoxir treatment induced statistical significant reduction of anhedonic behavior compared to vehicle treatment (p < 0.0001) and reversed depression-like phenotype in 90% of the rats (p = 0.0007), whereas Escitalopram only proved 57% efficacy. Moreover, Etomoxir revealed downregulation of interferon-*γ*, interleukin-17*α* and tumor necrosis factor-*α*. This indicate that alteration in metabolism is pivotal in the pathogenesis of depression, since CPT1 blockage is highly efficient in treating anhedonia and inflammation, thereby opening up for a novel class of antidepressant medication.

## Introduction

Major depressive disorder is a common and complex disease characterized by prolonged periods of suppressed mood and anhedonia, which is defined as loss of interest or pleasure in all or almost all activities^1–3^. Depression affects 350 million people worldwide and in 2030 predictions from WHO indicate that depression will be one of the largest causes of the disease burden globally^4^. The most commonly prescribed antidepressant treatment is selective serotonin reuptake inhibitors (SSRIs)^5, 6^. The antidepressant treatment used today has only shown moderate response rates of up to 50–60%^5^. This indicates that there is a large number of patients that respond inadequately to treatment, thereby underscoring the need for more effective treatment of depression. The pathology of depression has not been elucidated yet, but different theories have been proposed. This study focuses on a novel concept concerning upregulated lipid metabolism, based on several studies have shown an association between serum lipid concentrations and depression in patients^7–9^. A study by Chen *et al*. showed a significant reduction in serum triglycerides and high-density lipoprotein cholesterol in depressed patients^7^. This indicates that alteration in lipid metabolism is involved in the pathology of depression^7, 8, 10^.

Glucose and fatty acids are both used for cellular energy production during glycolysis and beta oxidation, respectively^11^. In beta oxidation, fatty acyl groups are transported from the cytosol and through the outer and inner mitochondrial membrane^12^. However, the impermeability of the mitochondrial membrane to acyl-CoA molecules necessitates the shuttling of acyl-CoA through the outer mitochondrial membrane by carnitine, which is catalyzed by carnitine palmitoyl transferase 1 (CPT1)^13^. CPT1 facilitates transfer of the acyl group from CoA to carnitine, thereby enabling transport of the acyl-carnitine through the outer mitochondrial membrane towards the mitochondrial matrix. Via the translocase the acyl-carnitine is transported to the matrix and there converted back to acyl-CoA catalyzed by carnitine palmitoyl transferase 2 (CPT2)^13^. Conversion of acyl-CoA to acyl-carnitine by CPT1 is the rate limiting step and CPT1 thus becomes a key regulator of the metabolism of the cell^12^. In diseases like cardiomyopathy, and psoriasis it has been shown that increased CPT1 expression is directly correlated with disease state^14, 15^. Although the exact role of CPT1 in depression is not known it is tempting to hypothesize that there is a link between CPT1 mediated lipid transport and depression due to its central role in cellular energy production from lipids and the fact that there is reduced lipid levels in depressed patients.

A potent blocker of CPT1, rate limiting for fatty acid metabolism and hence lipid metabolism, called Etomoxir is able to reverse the shift in metabolism, thereby favouring glucose metabolism as an energy source rather than lipids^16–18^. Etomoxir, a CPT1 antagonist, binds specifically to CPT1 and thus prevents the formation of acyl-carnitine, which is a necessary step for the transport of fatty acyl-CoA into the mitochondria^16^. This treatment regimen focusing on the metabolism is fundamentally very different from the most common treatment rationales of depression, which are generally based on the use of antidepressants stimulating monoaminergic transmission^5^. Under conditions with stress exposure metabolism shifts from using glucose, the primary energy source of the central nervous system, to fatty acids as energy source^12, 19–21^. Stress can be caused by internal or external events. Internal events include traumatic head injury and hormonal challenges, whereas external stressors encompass major adverse life events like bereavement or accumulation of minor stressors for example poverty, unemployment and family disharmony^6^. These different types of stressors formed the basis of establishing the chronic mild stress (CMS) model in rodents^22^. The CMS model is a state-of-art validated model mimicking depression by relying on the application of realistic stressors and thus incorporating the cardinal symptom, anhedonia, and thereby inducing a decrease in responsiveness to reward^1, 22^. The CMS protocol consists of exposure to a variety of stressors, e.g. food or water deprivation, tilting of cages, isolation or crowded housing, changing dark-light cycle etc., which results in behavioural deficits and decreased reward sensitivity^1^. Consequently, this leads to suppressed preference or consumption of a palatable sucrose solution, which therefore becomes a repetitive readout on the hedonic status of the rats and thereby becomes a rating measurement on the severity of the depression-like state^23^.

The purpose of the present study is to clarify the role of lipid metabolism on the development of depression by examining the effect of a blocker of lipid metabolism, Etomoxir, in the CMS model and compare it to a current standard treatment of depression, Escitalopram. Moreover, we want to examine the effect of Etomoxir on inflammation induced by a bacterial agent in human peripheral blood lymphocytes.

## Results

### Expression of carnitine palmitoyl transferase 1a mRNA in depressed patients

The expression of CPT1a mRNA in pathological brain samples of patients that committed suicide with a history of depression and compared non-depressed controls that committed suicide was analyzed by affymetrix analysis (Table 1). Results from this analysis showed a significant upregulation of CPT1a expression in cerebellum (p = 0.0021). There was no significant upregulation of CPT1a mRNA in the inferior temporal gyrus, nucleus accumbens and hippocampus (p = 0.0614, p = 0.0682 and p = 0.4831), although there was tendency of upregulation in these brain regions.

**Table 1:** mRNA expression of CPT1a.

Tissue	CPT1a expression (Control)	n	CPT1a expression (Depression)	n	Fold change	P-value
Cerebellum	5.96	10	16.55	10	2.78	0.0021
Inferior temporal gyrus	8.60	8	15.01	14	1.75	0.0614
Nucleus accumbens	6.12	7	10.93	13	1.78	0.0682
Hippocampus	10.25	9	11.91	13	1.16	0.4831

### Treatment of stress-induced depression by blocking carnitine palmitoyl transferase 1

**Figure 1:**
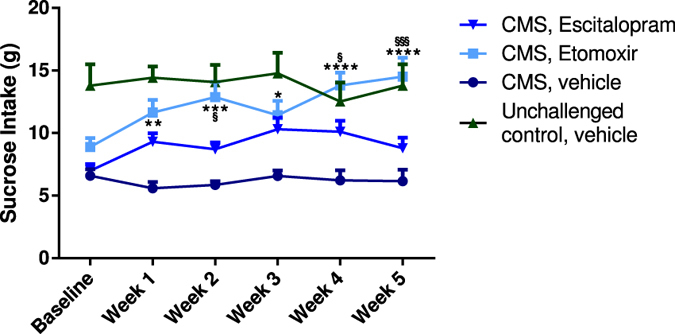
Comparison over time of the efficacy of Etomoxir and Escitalopram in chronic mild stress-induced depression. Baseline values indicate sucrose intake after four weeks of stress exposure. Sucrose intake in gram during five weeks of treatment with Escitalopram (n = 14, downwards triangle), Etomoxir (n = 20, square) or vehicle (n = 9, circle) with continuous chronic mild stress exposure. Unchallenged control group was not exposed to the stress protocol but received vehicle treatment (n = 9, triangle). All data are presented as mean ± SEM. The results from the repeated measures two-way ANOVA (interaction F = 2.31, DF = 15, p = 0.0043; time F = 2.21, DF = 5, p = 0.0535; treatment F = 13.63, DF = 3, p < 0.0001) with Tukey multiple comparisons post hoc test showed statistical significance between vehicle and Etomoxir (*), and Escitalopram and Etomoxir (§). Number of asterisks/paragraphs indicates level of statistical significance (*p = 0.01–0.05, **p = 0.001–0.01, ***p = 0.0001–0.001, ****p < 0.0001).

**Figure 2:**
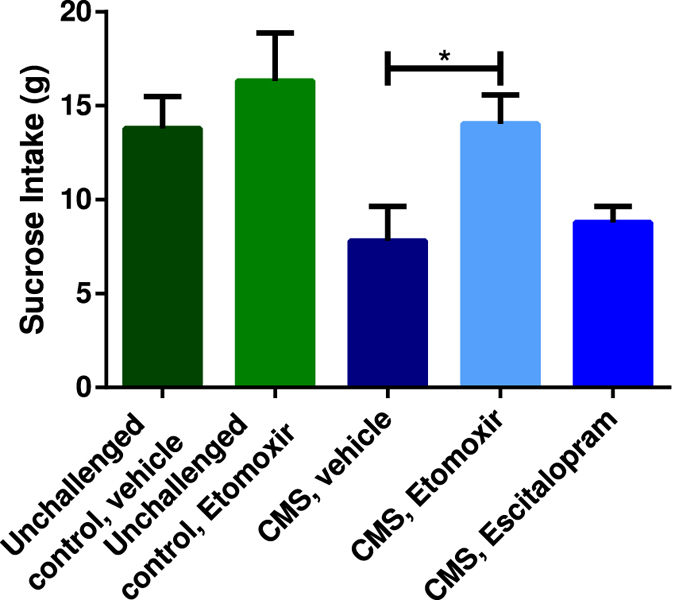
Treatment efficacy in the different groups after five weeks of drug treatment. All data are presented as mean ± SEM. The results from the one-way ANOVA (F = 4.178, DF = 4, p = 0.0052) with a Tukey multiple comparisons post hoc test showed statistical significance between CMS exposed animals receiving vehicle and Etomoxir. Number of asterisks indicates level of statistical significance (*p = 0.01–0.05).

**Figure 3:**
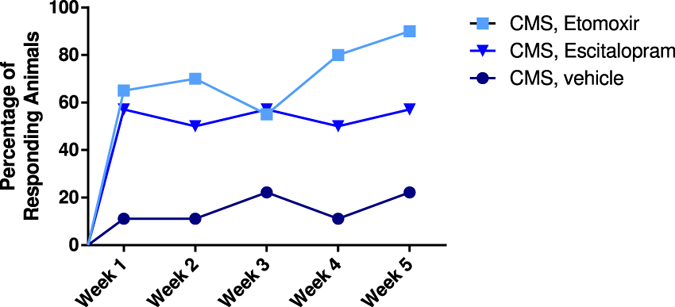
Percentage of responding animals in all five weeks of treatment. Etomoxir or Escitalopram administration reveal similar percentage of responding animals in week one and three, however treatment with Etomoxir shows higher percentage of responders in week two, four and five compared to Escitalopram.

**Figure 4:**
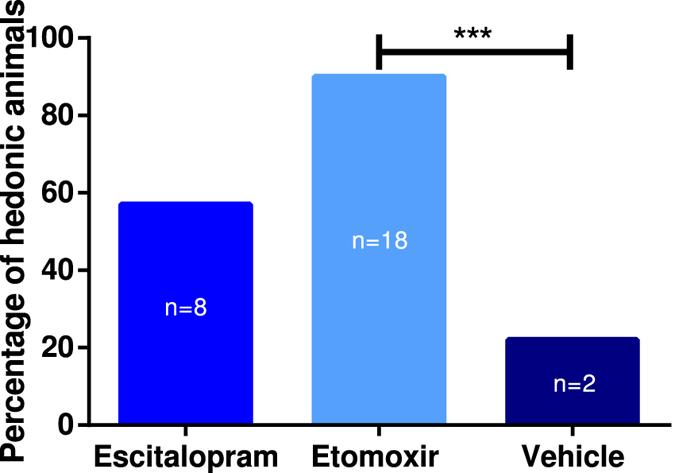
Percentage of hedonic animals after treatment with Escitalopram and Etomoxir. The results from Fischers exact test showed significant difference between treatment with Etomoxir (n = 20) and vehicle (n = 9)(***p = 0.0001–0.001). There was no statistical significance between Escitalopram (n = 14) and vehicle.

Rats were initially exposed to four weeks of CMS and subsequently exposed to stressors for another five weeks combined with drug or vehicle treatment (Fig. 1). The intake of sucrose solution was measured in order to determine the anhedonic status among rats. There was no significant difference between rats receiving vehicle treatment compared to groups receiving Escitalopram. In a separate group rats were treated with Etomoxir while exposed to stress for five weeks and compared to a vehicle group exposed to stress and an unchallenged control group. The intake of sucrose solution was significantly higher in rats treated with Etomoxir compared to the vehicle group in all five weeks (p = 0.0013, p = 0.0001, p = 0.0157, p < 0.0001 and p < 0.0001). Moreover, statistical significant difference was found between Escitalopram and Etomoxir treatment in week two, four and five (p = 0.0175, p = 0.0455 and p = 0.0004) revealing a significantly higher efficacy of Etomoxir compared to Escitalopram treatment. Treatment with Etomoxir during CMS exposure increased the level of sucrose intake to the same level as for unchallenged rats after five weeks of treatment. The total intake of sucrose was compared in all five groups after five weeks of treatment (Fig. 2). Statistically significant difference was found between rats exposed to stress treated with Etomoxir and CMS rats receiving vehicle (p = 0.0441). No significant differences were observed between unchallenged controls receiving vehicle or Etomoxir and stress exposed rats receiving Etomoxir. The percentage of animals responding over time receiving Etomoxir, Escitalopram or vehicle was compared (Fig. 3). The criterion for responders was set at an operational cut-off of 20% increase in intake of sucrose at the respective weeks compared to baseline prior to onset of treatment. The effect of treatment with Etomoxir and Escitalopram was almost the same in week one and three, whereas the percentage of responding rats to Etomoxir treatment was higher than Escitalopram treatment in week two, four and five. Treatment with Escitalopram showed 57% hedonic rats after five weeks of treatment with concomitant exposure to stress (Fig. 4). No significant difference was found in treatment efficacy of Escitalopram when comparing to vehicle. Etomoxir reversed the CMS sucrose drinking behaviour in 90% of the rats compared to baseline. In vehicle treated CMS exposed rats 22% recovered partly which was likely due to habituation to stressors. The difference in treatment efficacy between Etomoxir and vehicle treatment was statistically highly significant (p = 0.0007).

### The effect of carnitine palmitoyl transferase 1 downregulates blockage on the immune system

**Figure 5:**
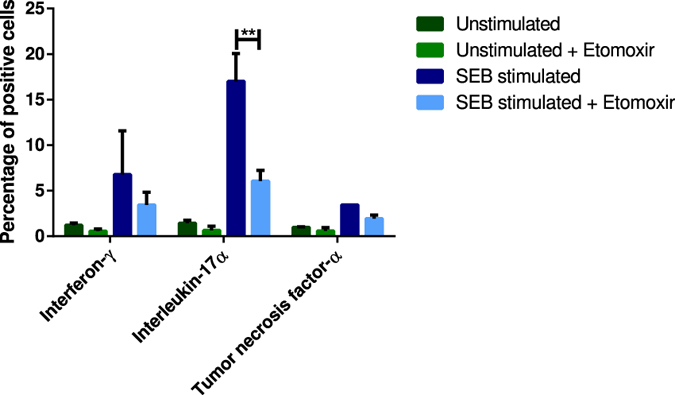
Human peripheral blood lymphocytes stimulated with staphylococcal enterotoxin B (SEB) for 48 h and gated for *CD*3^+^ cells. SEB stimulation activates the immune system. All data are presented as mean ± SEM. The results from the two-way ANOVA (interaction F = 3.576, DF = 6, p = 0.0287; cytokine F = 7.362, DF = 3, p = 0.0082; treatment F = 14,83, DF = 2, p = 0.0002) with a Tukey multiple comparisons post hoc test showed statistical significant difference in percentage of interleukin-17*α* positive cells between SEB stimulated cells and SEB stimulated cells receiving Etomoxir (**p = 0.001–0.01).

Human peripheral blood lymphocytes were stimulated with the T cell activating agent, staphylococcal enterotoxin b (SEB), for 48 hours in order to activate the immune system (Fig. 5). An unstimulated group and a SEB stimulated group were treated with Etomoxir. The human peripheral blood lymphocytes were gated for *CD*3^+^ cells. Unstimulated lymphocytes receiving Etomoxir and no SEB treatment showed low production of all cytokines measured. SEB stimulated lymphocytes receiving Etomoxir treatment revealed 49%, 64% (p = 0.0037) and 44% downregulation of interferon-*γ* (IFN-*γ*), interleukin-17*α* (IL-17*α*) and tumor necrosis factor-*α* (TNF-*α*).

## Discussion

The traditional way of thinking depression such as impairment in a single brain substructure or a single neurotransmitter system is no longer approved. Rather it has become evident that much more complex systems are underlying the depression pathology^24^. Imbalance in monoaminergic neurotransmission, impaired neurogenesis in hippocampus, high levels of glucocorticoids and inflammation in CNS are all well-founded theories describing the pathogenesis of depression^25, 26^. Established hypotheses may still be partly operative although they are incomplete in explaining the entire etiopathogenesis of depressive disorder and do not account for the inadequacy in conventional antidepressant treatment regimens either. Novel hypotheses, appreciating the etiology of the disease, are on demand for the development of more effective and safe antidepressants. Therefore, a new hypothesis involving upregulated lipid metabolism, as a consequence of persistent stress exposure, is potentially a main underlying driver and comprises possibly a missing link in understanding the pathophysiology of depression. This kind of consideration is a novel way of thinking the pathogenesis.

Lipids play an important role in normal neuronal functioning of the brain, as they are involved in signaling processes of the cell^27^. Endocannabinoids, a class of lipid mediators, are found in several tissues and involved in processes as neuronal signaling, immune responses, cell survival and apoptosis^28^. These endocannabinoids are thought to provide neuroprotection via cannabinoid receptors. Lipids, as arachidonic acid and docosahexaenoic acid, constitute approximately 20% of lipids in the brain, and therefore essential for maintenance of a normal brain metabolism^29, 30^. Imbalance in the composition of these important lipids is associated with several brain diseases^29^. Derivatives of arachidonic acid, e.g. N-arachidonoyl-dopamine, activates the cannabinoid type-1 receptor resulting in increased dopamine release^28, 31^. Both dopamine and cannabinoid neurotransmission is involved in emotional and motivational behavior^28, 31–35^. With an upregulated lipid metabolism follows a decreased level of serum lipids, which is a finding associated with depression^7, 8^. CPT1 is a key enzyme in lipid metabolism as it catalyzes the rate-limiting step in beta oxidation^12, 36^. We found a significant upregulation of CPT1a mRNA levels in cerebellum of people with history of depression which committed suicide, compared to healthy controls. This suggests an association between CPT1a upregulation and depression, as a consequence of upregulated lipid metabolism. Liu *et al*. concluded that network alterations in cerebellum is associated with cognitive emotional impairments in depression^37^. We also found CPT1a mRNA upregulation in inferior temporal gyrus, nucleus accumbens and hippocampus, though these upregulations of CPT1a mRNA were not statistically significant different compared to controls. Additionally, two ethnic populations, called Hutterites and Inuits, both living in Canada have two different mutations in CPT1a, which either makes the protein dysfunctional (Hutterites) or decreases the activity to 22% (Inuits) (Table 2)^38–42^. In these populations the life time prevalence rates of depression is 0.35% and 3%, respectively, which is rather low compared to the rate in the gross Canadian population at 16%^43, 44^. Whether these low rates of depression is caused by genetic or environmental alterations is unknown, however, the finding of CPT1a mutations in the Hutterite and Inuit population supports the novel theory in this study stating that a decreased activity of CPT1 increases resiliency for developing depression^45^.

**Table 2:** Carnitine palmitoyl transferase 1a (CPT1a) mutations in humane populations.

	CPT1a mutation	% CPT1a activity	% people with mutation	Depression rates
Canadian population	Wild type	100%^42^	~0%^39^	16%^44^
Hutterites	2129 G to A → AA710 Gly to Glu	~0%^38–40, 42^	60%^39^	0.35%^43^
Inuits	1436 C to G → AA479 Pro to Leu	22%^41, 42^	98%^57^	3%^44^

Blocking the lipid metabolism by Etomoxir, a CPT1 antagonist, we therapeutically downregulate the activity of CPT1, and thereby also CPT1a. This highlights a new mechanism of action for novel antidepressant medication, which is examined in this study using the CMS model. Studies suggesting alterations in fatty acids and lipid metabolism demonstrated antidepressant effects of l-acetyl-carnitine treatment in mice^46, 47^. This treatment is comparable with blockage of CPT1a, since this also leads to increased acetyl-carnitine levels. However, Etomoxir treatment has in addition anti-inflammatory effects. This result underpins the theory and findings of this study regarding blockage of lipid metabolism as a potent antidepressant treatment strategy. Rats exposed to the CMS model, thus developing anhedonic behavior, received Etomoxir and showed significant increased intake of sucrose in all five weeks compared to the vehicle group. Moreover, the response rate of Etomoxir was higher than the response rate of Escitalopram, as 90% of the animals responded to Etomoxir treatment, while only 10% were classified as non-responders. Treatment with Etomoxir resulted in a statistically significant higher percentage of responding animals compared to vehicle treatment showing only 22% healthy rats after five weeks of treatment. All animals from each group were included, therefore there was no significant effect of Escitalopram. When analyzed at an individual basis 60% of the Escitalopram treated animals were classified as drug-responders, which is in the range that we have observed repeatedly in previous studies^2^. Comparison of sucrose intake after five weeks of treatment in all groups demonstrated that treatment with Etomoxir was significantly different from vehicle treatment. Moreover, the intake of sucrose after Etomoxir treatment was almost equal to the sucrose intake of unchallenged rats indicating that Etomoxir is able to reverse the anhedonic behaviour of rats exposed to stressors. The principle underlying Etomoxir treatment strategy is dramatically different from that of SSRIs, which increases the level of extrasynaptic serotonin. A disadvantage of conventional antidepressants is slow onset of antidepressant action^6^. Thus it takes several weeks before SSRIs are therapeutically effective^48^. Etomoxir treatment showed major effects after week one with a response rate of approximately 70% and 90% after five weeks of treatment. Additionally, Etomoxir revealed a significant higher percentage of responding animals in all five weeks compared to Escitalopram and vehicle.

Studies have suggested a correlation between depression and immune dysregulation as increased levels of pro-inflammatory cytokines were found in depressed patients compared to healthy subjects^25, 26, 49^. It has been shown that increased activity of CPT1a results in increased inflammatory and memory T cell responses^50^. Therefore, inhibition of CPT1 by Etomoxir is predicted to decrease the inflammatory response. This was supported by data from this study, in which human peripheral blood lymphocytes were stimulated with SEB, a bacterial antigen, in the presence or absence of Etomoxir. Treatment with Etomoxir mediated downregulation of cytokines IFN-*γ*, IL-17*α* and TNF-*α* of 49%, 64% and 44%, respectively, which were upregulated by SEB stimulation. These have, among others, been suggested to be involved in the pathogenesis of depression although the exact pathophysiological mechanism is still unknown^51^. Several studies have demonstrated inflammatory changes in the brain of depressed patients^52–54^. Moreover, a study regarding the experimental autoimmune encephalomyelitis model of multiple sclerosis found that treatment with Etomoxir revealed reduced demyelination of neurons and reduced infiltration of immune cells in the CNS thereby counteracting inflammatory responses^12^. All these findings indicate that Etomoxir has anti-inflammatory effects and lipid metabolism plays a role in supporting the inflammatory response within the CNS^12^. The data presented in this study has motivated us to propose a new hypothesis of depression suggesting that the pathophysiology of depression is a combination of both dysregulated lipid metabolism and dysregulated immune response.

We demonstrated that inhibition of CPT1 by systemic application of Etomoxir has beneficial effects in the treatment of depression in a highly validated CMS depression model. The model has unique predictive validity in antidepressant drug screening, essentially without any false positives. Moreover, treatment with Etomoxir showed significantly higher intake of sucrose compared to Escitalopram and vehicle, and also higher response rate of up to 90%. We also demonstrated an anti-inflammatory effect of Etomoxir as the levels of cytokines, IFN-*γ*, IL-17*α* and TNF-*α*, were downregulated compared to controls. Treatment with Etomoxir and thereby blocking the lipid metabolism paves the way for rethinking strategies in the development of novel treatment regimens of depression.

## Methods

### Affymetrix Analysis

Tissue was isolated from people that committed suicide. The CPT1a expression analysis was performed on tissue selected from either patients with history of depression or non-depressed controls. Tissue from patients or healthy donors has been isolated according to Gene Logic protocols. Ethical and medical approvals were obtained by Gene Logic, Inc. 708 Quince Orchard Rd. Gaithersburg, MD 20878. Afterwards the mRNA was purified and analyzed for expression of CPT1a in affymetrix analysis (Affymetrix ID 203634_s_at and 210688_s_at) Genbank ID: NM_001876, and expression was analyzed with GeneExpress® and e-Northern^TM^ proprietary informatics programs of Gene Logic.

### Animals

The animal experiment was conducted according to NIH guidelines and was approved by the Danish National Committee for Ethics in Animal Experimentation (2008/561-477). Male Wistar rats purchased from Taconic, Denmark, were used for the CMS model. The weight of the rats was approximately 200 g when the experiment was initiated and approximately 350 g when stress exposure was initiated. The rats were housed singly with 12 h light/dark cycle and food and water was available ad libitum except when these parameters were applied as stress inducers. The following paragraphs concerning the CMS model was performed according to the protocol by Jayatissa *et al*.^55^.

### Chronic Mild Stress protocol

The animals were divided into two groups; one group was exposed to stress and one control group was unchallenged. The two groups were matched in such a manner that both mean and standard deviation in sucrose consumption were similar. The animals were then placed in separate rooms. One group was exposed to an initial four week period of chronic mild stressors, while the other group was left undisturbed. Food and water was freely available for the unchallenged group, except 14 h before the sucrose consumption test where the animals were food and water deprived. The stress paradigm persisting in four weeks involved one period of intermittent illumination, stroboscopic light, grouping of the animals, and food and water deprivation. Moreover, there were two periods with no stress and soiled cage and three periods of tilting the cage 45°. Stressors were exchanged every morning and night.

### Sucrose consumption test

In order to quantify the hedonic state of the animals a sucrose consumption test was performed. The animals were trained in five weeks in order to consume a palatable sucrose solution. In the period of the five weeks training, the animals were tested twice a week during the first three weeks and only once a week the last two weeks. The animals were deprived for food and water in 14 h before the sucrose consumption test. The test involved 1 h access to a 1.5% sucrose solution in a one bottle paradigm. When the stress period was initiated the sucrose consumption test was performed once a week. A cut-off of 20% increase in sucrose intake, compared to baseline, at the respective week was applied to define responders versus non-responders. To ensure that basic thirst was similar for all groups diurnal water intake was measured in the beginning of each week.

### Drug administration

After four weeks of CMS exposure, the animals were treated with either drug or vehicle for five weeks while still exposed to stressors. Etomoxir (Meta-IQ ApS, Denmark), a specific CPT1 inhibitor^56^, was heated to 37 °C and dissolved in sunflower oil. Etomoxir was administered intraperitoneally every day in a dosage of 4 mg/kg. Escitalopram (Lundbeck, Denmark) was dissolved in saline and was administered intraperitoneally daily in a dosage of 5 mg/kg. The vehicle group received daily injections with sun flower oil intraperitoneally. All injections were administered in the morning and on Fridays subsequent to the sucrose test.

### Intracellular staining for flow cytometry

Experiments involving human blood samples were carried out in accordance with the approved guidelines and regulations according to the Declaration of Helsinki. Informed consent forms of these donors have been obtained. The study and use of human blood material was approved by Ethical Committee for Region North Denmark (N-20150073). Blood lymphocytes were isolated from humans using a buffy coat. Sodium heparin full blood was centrifuged at 2000 g for 15 min and the white blood cell layer was harvested. To ensure harvesting of all white blood cells, the cells were centrifuged over a Ficoll density gradient at 2000 g for 10 min. The white blood cells were then harvested, centrifuged at 2000 g for 5 min and the supernatant was discarded. The cells were plated with a density of 2 × 10^6^ cells/well in 6-well plates and grown for 48 h. The cells were cultured in RPMI medium (cat#61870-010, Gibco, CA, US) containing 10% fetal calf serum (cat#10270-106, Invitrogen, CA, US) and 1% penicillin/streptomycin (cat#15140-122, Life Technologies, CA, US) in the presence or absence of staphylococcal enterotoxin B (30 ng/ml) (cat#S4881, Sigma Aldrich, MO, US). Some cells were treated with Na-Etomoxir (100 *μ*M) (cat#E1905, Sigma Aldrich, MO, US). After 48 h, the cells were re-suspended in phosphate buffer saline (PBS)/bovine serum albumin (BSA) (cat#EQBAH62-00, Europa Bi-products, UK), collected in tubes and centrifuged at 1500 g for 4 min. The supernatant was discarded and PBS/BSA was added. The cells were plated into a 96-well plate, centrifuged at 1500 g for 4 min and the supernatant was aspirated. The cells were stained for APC mouse anti-human CD3 (cat#561810, BD Biosciences Pharmingen, CA, US) or PerCP-Cy mouse anti-human CD4 (cat#560650, BD Biosciences Pharmingen, CA, US) diluted in PBS/BSA and incubated on ice for 1 h. The staining procedure was carried out according to Intracellular Staining Kit (cat#ANN0001, Intracellular Staining Kit, Invitrogen, CA, US). The cells were washed in PBS containing 0.5% BSA and centrifuged at 1500 g for 4 min twice, after which the cells were fixed in IC fixation buffer (cat#FB001C, BD Biosciences Pharmingen, CA, US) at 4 °C for 10 min. After washing two times in IC permeabilization buffer (cat#PB001C, BD Biosciences Pharmingen, CA, US), the cells were centrifuged at 1500 g for 4 min and the supernatant aspirated. The antibodies FITC mouse anti-human IFN-*γ* (cat#561057, BD Biosciences Pharmingen, CA, US), PE mouse anti-human IL-4 (cat#562046, BD Biosciences Pharmingen, CA, US), mouse anti-human IL-8 (cat#340509,BD Biosciences, Becton Dickinson Company, CA, US), PE mouse anti-human IL-17*α* (cat#560438, BD Biosciences Pharmingen, CA, US) and FITC mouse anti-human TNF-*α* (cat#562082, BD Biosciences Pharmingen, CA, US) diluted in permeabilization buffer were added and incubated on ice for 30 min. The cells were washed twice in permeabilization buffer and centrifuged at 1500 g for 4 min and lastly re-suspended in PBS for further analysis using flow cytometry.

### Statistical analysis

All data are presented as mean ± SEM. P values less than 0.05 were considered significant. The sucrose data were analysed by using repeated measures two-way ANOVA and one-way ANOVA with a Tukey multiple comparisons post hoc test. Differences in number of animals responding to treatment were analysed by Fishers exact test. The data concerning human peripheral blood lymphocytes were analysed by a two-way ANOVA with a Tukey multiple comparisons post hoc test.
